# Application of Microsatellites to Trace the Dairy Products Back to the Farm of Origin

**DOI:** 10.3390/foods12224131

**Published:** 2023-11-15

**Authors:** Simona Perga, Cristina Biolatti, Isabella Martini, Francesco Rossi, Alfredo Benso, Pier Luigi Acutis, Alessandro Bagnato, Domenico Cognata, Piero Caroggio, Simone Peletto, Paola Modesto

**Affiliations:** 1 Istituto Zooprofilattico Sperimentale del Piemonte, Liguria e Valle d’Aosta, 10154 Turin, Italy; simonaperga77@gmail.com (S.P.); cristina.biolatti@gmail.com (C.B.); isabella.martini@izsto.it (I.M.); pierluigi.acutis@izsto.it (P.L.A.); simone.peletto@izsto.it (S.P.); 2 Computer and Control Engineering Department, Polytechnic of Turin, 10100 Turin, Italyalfredo.benso@polito.it (A.B.); 3 Department of Veterinary and Animal Science, Università degli Studi di Milano, 26900 Lodi, Italy; alessandro.bagnato@unimi.it; 4 Azienda Sanitaria Locale BI, 13900 Biella, Italy; domenico.cognata@aslbi.piemonte.it; 5 Azienda Sanitaria Locale 1 Imperiese, 18100 Imperia, Italy; p.caroggio@asl2.liguria.it

**Keywords:** genetic traceability, dairy products, STR

## Abstract

The increasing number of food frauds, mainly targeting high quality products, is a rising concern among producers and authorities appointed to food controls. Therefore, the development or implementation of methods to reveal frauds is desired. The genetic traceability of traditional or high-quality dairy products (i.e., products of protected designation of origin, PDO) represents a challenging issue due to the technical problems that arise. The aim of the study was to set up a genetic tool for the origin traceability of dairy products. We investigated the use of Short Tandem Repeats (STRs) to assign milk and cheese to the corresponding producer. Two farms were included in the study, and the blood of the cows, bulk milk, and derived cheese were sampled monthly for one year. Twenty STRs were selected and Polymerase Chain Reactions for each locus were carried out. The results showed that bulk milk and derived cheese express an STR profile composed of a subset of STRs of the lactating animals. A bioinformatics tool was used for the exclusion analysis. The study allowed the identification of a panel of 20 markers useful for the traceability of milk and cheeses, and its effectiveness in the traceability of dairy products obtained from small producers was demonstrated.

## 1. Introduction

The free movement of safe food is an essential aspect of the European Union’s (EU) internal market and trade and contributes significantly to the health and well-being of citizens and to their social and economic interests. According to EU law, food fraud is an intentional action for the purpose of deceiving consumers and obtaining a financial gain. Estimates on the economic impact of food fraud vary significantly among authors, from a cost of $10–15 billion to $40 billion, and others suggesting a value of at least $65 billion globally [1,2,3]. In the last decade, the prevention of food fraud has increasingly been a focus of EU policy, agri-food industries, and government authorities responsible for ensuring food quality and safety. The Directorate General for Health and Food Safety (DG SANTE) cooperates with the European Anti-Fraud Office (OLAF) for European Member States to ensure that food is sustainable and safe. Competent authorities of the Member States are required to detect violations of the rules governing the agri-food chain and also to identify possible intentional violations of those rules, perpetrated through fraudulent or deceptive practices [4]. Food fraud represents a relevant issue also in Italy and it mainly concerns meats, wines, dairy products, and high-quality products. According to the annual report of the ICQRF (Italian Central Inspectorate of Quality Protection and Fraud Repression) in 2022, 55.735 products were inspected and agri products accounting for 32 million euros were seized [5].

In this context, an effective control system represents the best guarantee to protect the safety and the quality of production and contributes to safeguarding the economic value of products on the markets (especially traditional and high-quality foods such as those with a protected designation of origin—PDO—and protected geographical indications—PGI).

Dairy products, particularly milk, rank among the top 10 food categories with the most reported instances of food fraud [6,7,8]. Among the 5890 cases of food fraud reported in the HorizonScan global database between 2000 and 2018, 4% (245) reported cases are fraud of dairy product [6].

Dairy products also represent a high source of food-borne disease; therefore, dairy product fraud can have not only an economic impact but also implications on the health of consumers [6,9]. The traceability of traditional cheeses produced in small farms is a challenging issue. It seems feasible that in order to increase the product supply, the amount of sold cheese does not originate from authorized farms. Since nothing is known about the hygienic conditions of the unauthorized farms, consequences on consumer health could occur. Gimonkar et al. (2021) reported that between 2000 and 2018, the majority of dairy fraud cases were due to fraudulent documentation (51%), adulteration or substitution of dairy products or their ingredients (32%), and the production and manufacturing of a dairy product in “Unapproved premises” (11%), which include producers supplying milk and dairy products without authorization from the health authority [6].

Given the general interest and importance of the topic, new methods for the authentication of these products are needed [6,10,11]. A wide variety of analytical methods for food traceability and authentication have been developed and tested [9,12]. These techniques can be grouped into chemical/biochemical and molecular techniques [2,13]. Each method enables us to obtain information on the composition and characteristics such as geographical origin, presence of adulterants, and species or varieties used in the food production process [14].

Molecular traceability methods are currently applied for the identification of fraud and traceability and authentication of food products [6,9,10,11]. They also play an important role in guaranteeing the origin and safety of food products and in supporting the development of areas with a marginal economy through the authentication of typical and niche products [13,14,15,16]. Among several molecular traceability systems, genetic methods are characterized by high efficiency, specificity, and sensitivity; moreover, they are applicable to different food matrices and processed products.

While several molecular methods have already been developed for species/breed detection in dairy products [6,16,17,18,19,20,21,22], the application of these methods for the traceability of the origin (farm or producer) in traditional dairy products has not been investigated due to technical process conditions.

The genetic traceability of dairy products poses some technical challenges as cheese comes from bulk milk (BM), which contains DNA from different cows of the farm and from different lactation periods and undergoes consistent changes during manufacturing processes, such as ripening, that may compromise the quality and quantity of the extracted DNA, making it unsuitable for subsequent analyses [22].

Currently, no studies in the literature have focused on the development of biomolecular methods which would be useful for attributing dairy products to the farm of production. The aim of this study was to set up a genetic tool for tracing the farm of origin. In particular, we investigated the use of microsatellites (STRs) to identify in the BM and in the derived cheese (CHE) a “DNA fingerprint” of the farm of origin which would be useful to assign dairy products to the corresponding producers.

Although DNA pooling is a technique already proposed as a method to reduce analysis costs in quantitative and population genetic studies [23,24,25,26,27], this technique has never been applied before for the molecular traceability of dairy products (BM and CHE), which can be considered natural pools of DNA.

Given the absence of similar studies and the difficulties related to the matrix, method, and interpretation of the results, we decided to conduct this study under controlled conditions, developing the protocol and evaluating its effectiveness in small farms.

Results showed that BM and derived CHE express an STR profile composed of a subset of STRs of the lactating animals. A bioinformatics tool was used for the exclusion analysis. The study enabled the identification of a panel of 20 markers which would be useful for the traceability of milk and cheeses.

## 2. Materials and Methods

### 2.1. Sample Collection

Two farms were included in the study. The first farm, located in the province of Biella (BI) (Piedmont, Italy), was composed of 12 cows belonging to the Pezzata Rossa d’Oropa breed, and the second farm, located in the province of Imperia (IM) (Liguria, Italy), hosted 14 Bruna Alpina breed cows. The two farms were chosen considering: (i) a low number of animals (between 10 and 20); (ii) the farmer’s openness to produce cheese with the BM of the sampling day, to store it separately from the rest of the production for 60 days, and finally to sell it for research purposes; (iii) localization in traditional cheese production areas where cheese traceability could be an issue.

Samples were collected by official veterinarians of the Local Health Authority. Blood samples were collected once during the annual health testing of the animals, and milk samples of each cow (M) were collected once at the beginning of the study. BM and derived CHE were sampled monthly for one year. The producers made cheese with the BM produced on the sampling day and the cheese ripening period was 60 days.

Blood samples were collected using Vacutainer tubes containing Ethylenediaminetetraacetic acid (EDTA) and stored at −20 °C until further analysis.

M and BM samples were processed for isolation of somatic cells immediately after they arrived in the lab (40 mL for each M and 50 mL for BM). Samples were centrifuged at 841× *g* for 10 min (min) at 4 °C. The cell pellet was washed twice with PBS and centrifugation at 473× *g* for 2 min was performed after each washing. Then, the pellet was placed in a 1.5 mL Eppendorf and centrifugated at 21,250× *g* for 3 min. PBS was removed after the centrifugation, and the dry pellet was stored at −20 °C until DNA extraction.

### 2.2. First Markers Selection

In collaboration with the Department of Veterinary Sciences and Technologies for Food Safety (VSA), University of Milan, a group of 62 STRs was selected from a panel of 280 already tested on Brown cattle (Table 1) [26]. The selection was made according to some criteria: (1) high polymorphism and absence of null alleles in Brown Cattle breed; (2) amplification length between 90 and 300 bp; (3) lack of linkage disequilibrium.

### 2.3. Genotyping Protocol Set Up: DNA Extraction and STR Amplification

Commercial DNA purification kits were used to isolate DNA from the different samples based on matrix type, according to the manufacturer’s instructions. Pure Link^TM^ Genomic DNA Mini Kit (Thermo Fisher Scientific, Waltham, MA USA) was used for blood and somatic cells samples. For DNA extraction from CHE, the QIAamp Fast DNA Stool Mini Kit (Qiagen, Hilden, Germany) was used with some modification: 200 mg of each CHE sample was aliquoted and homogenized by beads in lysis buffer using tissue grinding tubes and incubated at 70 °C for 5 min. The producer protocol of extraction was then used. The concentration of DNA extracted from all three matrices was checked using NanoDrop ND-1000 spectrophotometer (NanoDrop Technologies, Wilmington, DE, USA) and samples were stored at −20 °C until use.

For each STR, an end-point PCR was performed to evaluate the correct amplification of each locus. Each PCR was performed in a total volume of 25 μL containing 1X Q solution, 1X PCR buffer, 1.5 mM MgCl_2_, 0.4 mM dNTPs, 1 UI of Taq DNA polymerase (Hot StartTaq DNA polymerase Kit, Qiagen), and 0.5 μM of each primer (Thermo Fisher Scientific). From 20 to 100 ng of DNA extracted from blood (BI = 7, IM = 5) was analyzed. A negative control containing no template was added at each run to ensure the absence of contaminations. The thermal cycling conditions were initial Taq activation step at 95 °C for 15 min followed by 45 cycles of denaturation at 95 °C for 30 s (sec), primer-specific annealing temperature for 45 s, extension at 72 °C for 1 min, followed by a final extension at 72 °C for 30 min. PCR products were displayed by electrophoresis on 2% (*w*/*v*) agarose gel. In order to verify their specificity, amplified DNA products with unexpected length were purified from agarose gel using Illustra^TM^, AutoSeq^TM^ G-50 Dye Terminator removal kit (Ge Healthcare, Chicago, IL, USA), and sequenced by Big-Dye Terminator kit v.3.1 (Thermo Fisher Scientific), according to the manufacturer’s instructions. Capillary electrophoresis was performed using 3130 Genetic Analyzer (Thermo Fisher Scientific). The sequences obtained were compared with the sequences deposited in Genebank through BLAST software (https://blast.ncbi.nlm.nih.gov/Blast.cgi (accessed on 18 August 2023)) using the blastn algorithm. A reduced panel of STR was selected according to the following parameters: (1) amplification and presence of polymorphism in the analyzed samples; (2) amplification length between 90 and 300 bp.

### 2.4. Final STR Markers Selection and Analysis

The panel of 50 STRs selected in the first step was tested by end-point PCR on DNA samples extracted from six cheeses from both BI and IM farms. Each PCR reaction was performed in a total volume of 25 μL containing 1X Q solution, 1X PCR buffer, 1.5 mM MgCl_2_, 0.4 mM dNTPs, 1 UI of Taq DNA polymerase (Hot StartTaq DNA polymerase Kit, Qiagen), and 0.5 μM of each primer (Thermo Fisher Scientific). From 20 to 100 ng of template were analyzed. A negative control containing no template was added at each reaction to ensure the absence of contaminations. The amplification profile was carried out as follows: initial denaturation at 95 °C for 15 min, followed by 45 cycles at 95 °C for 30 s, primer-specific annealing temperature for 45 s, and extension at 72 °C for 1 min, followed by final extension at 72 °C for 30 min. PCR-amplified products were displayed by electrophoresis on 2% (*w*/*v*) agarose gel. Amplified DNA products with unexpected length were sequenced and analyzed as described in the previous paragraph. The sequences obtained were compared with the sequences deposited in Genebank through BLAST software using the blastn algorithm.

The STRs successfully amplified in the two different matrix samples showing the variable length of amplification products among subjects were selected. The forward primers were labeled with the FAM or HEX fluorophore (Table 1) in order to be detected by the capillary electrophoresis automatic DNA sequencer 3130 Genetic Analyzer (Thermo Fisher Scientific).

### 2.5. Genotyping of Subjects and Analysis of BM and CHE

The selected STRs (Table 1) were amplified by end point PCR in all samples collected. Each PCR reaction was performed in a total volume of 10 μL containing 10 to 30 ng of genomic DNA, 1X PCR buffer with 1X Q solution, 1.5 mM MgCl_2_, 0.4 mM dNTPs, 0.5 μM of each primer (Eurofins Genomics Europe, Ebersberg, Germany), and 1 UI of Taq DNA polymerase (Hot StartTaq DNA polimerasi Kit, Qiagen). A control not containing DNA was amplified in each run to assess the absence of contaminations. The thermal cycling conditions were initial inactivation at 95 °C for 15 min, followed by 45 cycles at 95 °C for 30 s, annealing at 54 °C for 45 s and extension at 72 °C for 1 min, followed by final extension at 72 °C for 30 min. The annealing temperature was set at 56 °C for the reactions containing the STRs MB064_HBB and BMS4044. Each sample was tested three times.

The analysis of the amplification products was performed by capillary electrophoresis using ABI PRISM 3130xl Genetic Analyzer (Thermo Fisher Scientific) and POP-7TM separation polymer. GeneMapperTM v. 5 software and size standard ROX 500^TM^ (Thermo Fisher Scientific) were used to assign the allele size (AS).

In order to guarantee the absence of conditioning in the assignment of AS, no information was given to the reader operator regarding the composition (number and genotyping profile of animals) of the GO (group of origin from each farm) during the evaluation of the BM and CHE samples. Some samples were performed several times to verify the attribution agreement of the AS. The AS was attributed only to alleles amplified with a high degree of uncertainty.

Genotyping data were analyzed with FSTAT version 2.9.4 software to estimate and test the genetics parameters of the two GOs [28].

### 2.6. Evaluation of Dairy Products Traceability Protocol

To demonstrate the ability of the selected panel to trace the origin of BM and CHE, we decided to implement an algorithm able to verify the correlation between the fingerprinting of the product (BM or CHE) and the genetic characteristics of the GO. The algorithm was developed in collaboration with the Polytechnic of Turin; details can be found in Rossi et al. (2018) [29].

The developed algorithm verified how much the “fingerprinting” of the product (BM or CHE) is correlated with the genetic characteristics of the cows included in the group of production. Based on the profiles of each animal of the farm, the software created an ideal linear combination corresponding to the “fingerprinting” of the farm. It then compared this combination with the result of the BM or CHE analysis and attributed a correlation value, indicative of the actual origin of the sample from the candidate group of animals. In particular, the analysis was divided into two separate and complementary parts: the test step and the simulation step. In the first step, the presence of all BM and CHE alleles is checked in the GO alleles set and a “penalty score” is produced that represents an increasing value proportional to the number of unmatching between BM and/or CHE and the GO. In the simulation step, the evolutionary algorithm generates a linear combination of cows in the farm, which correlates as much as possible with the signature of BM and/or CHE. The more the penalty score tends to zero, the more an appropriate match between the dairy products and the cows’ group exists, and the simulation step could be considered reliable (and *vice versa*). The threshold value according to which the correspondence between LM or FOR and the GO can be rejected may be low (restrictive approach) or high (more tolerant approach). The latter case can be applied, for example, for supposed errors in reporting the number and identity of the cows included in the GO.

The simulation process is based on the hypothesis that if an estimation of quantities of the alleles exists (i.e., the peaks heights in Relative Fluorescence Units-RFU), it is possible to obtain a linear combination of cows to recreate the “signature” of the BM or CHE, according to a group of cows which have produced BM or CHE. The value obtained by the simulation process ranges from 0 to 1 (with the best correlation equals to 1) and can be used as a further index to be added to the penalty score when attributing the origin of the product to the GO. The hypothetical score equal to zero and correlation equal to one is the best case of attribution.

The genotyping data of each farm and the respective BM and CHE obtained using our STR panel were analyzed with the developed algorithm in order to verify the possible attribution and/or exclusion of the origin of the products. Then, a cross-analysis was performed by testing the correlation between the data of the dairy products of one farm and those of the GO signature of the other producer.

## 3. Results

### 3.1. Sample Collection

Twenty-six EDTA blood and individual milk from the BI (n = 12) and IM (n = 14) farms and twenty-three samples of bulk milk (BI n = 12 IM = 11) were collected. All samples were uniquely identified and processed as described above and then stored at −20 °C until further analyses.

Twenty-three cheese samples were collected: 11 from IM and 12 from BI farm. All cheese samples were aliquoted in a 2 mL tube and stored at −20 °C.

The number of animals involved in the production of BM and CHE in each sampling varied from a minimum of four to a maximum of seven for the BI farm and from a minimum of nine to a maximum of fourteen for the IM ones.

### 3.2. STR Genotyping Protocol Set Up and Marker Selection

DNA was extracted from all collected samples. The quantity of extracted DNA from the milk samples resulted in quantities between 6.5 and 25.5 ng/µL with quality values, calculated on the basis of the 260/280 and 260/230 ratio, between 1 and 1.6 in the first case and 0.8 and 1.9, respectively. The DNA extracted from the cheese samples resulted in quantities between 3.5 and 19.5 ng/µL with quality values between 1 and 1.6 (260/280) and 0.2 and 0.5 (260/230).

In the first phase, an efficient method for the genotyping of the individuals included in the study starting from DNA obtained by blood (BI = 7, IM = 5) was set up. Fifty STRs were initially selected according to the cited parameters. Markers showing no amplification or a single fragment in all samples analyzed were excluded. Detailed information on microsatellites (chromosome assignment, size and number of alleles detected in the Brown breed, and sequence of the primers used for the analysis) is shown in Table 1.

The STRs were analyzed by end-point PCR using BM and CHE samples (six different samples each) and the developed protocol. Markers showing no amplification in one or two different matrices were excluded in subsequent analyses.

Among the analyzed STRs, BM0143 and INRA133 markers produced fragments with greater and shorter lengths than expected, respectively, in all samples. Sequencing analysis confirmed the markers’ specificity and the presence of microsatellites. The INRA133 marker produced an unspecific fragment amplification, shorter than 200 bp, that was not considered when genotyping the samples.

Within the group of 50 STRs, 20 markers were selected (Table 1). The forward primers of each marker were labeled with the FAM or HEX fluorophore in order to be analyzed by the capillary electrophoresis with an automatic 3130 Genetic Analyzer (Thermo Fisher Scientific).

The selected markers showed: (1) high peak definition and characteristic shadow bands in the electrophoretic trace; (2) absence of null alleles; (3) robustness of the amplification reaction; (4) size of the amplification product shorter than 300 bp; (5) high polymorphism.

### 3.3. Genotyping of Subjects and Analysis of BM and CHE

All blood samples from the two farms were genotyped and each analysis was replicated three times. IM and BI samples showed variable number of alleles, with some alleles shared and others specific to each farm (Table 2).

In order to verify the feasibility of the individual milk instead of the blood sample for the genotyping, seven samples from BI and nine samples from IM were randomly chosen and tested by six markers (AGLA29, BM3507, BMS0607, MB025, SRC276, Z27077) with the developed protocol. Individual milk genotyping showed the same results obtained by blood analysis.

All BM and CHE samples were tested with the 20 STR markers, and three replicates for each sample were set up. An allelic profile was not obtained in 24 out of 480 cases (5%) for BM and in 29 out of 460 cases (6.3%) for CHE samples.

The markers with the highest number of non-genotyped samples (five each) were BM3507, INRA133, and BMS0607. The genotyping of all samples (blood, BM, CHE) detected a total of 109 and 119 alleles in the LM of the BI and IM farm, respectively. In the BI CHE and IM CHE samples, 119 and 122 alleles were detected, respectively (Table 3). For BM samples, the markers with the highest number of detected alleles were HUJ625 (BI farm) and BM720 (IM farm). For CHE samples, the markers with the highest number of detected alleles were BM720 and BMS0607 in the IM farm, and AGLA232 and HUJ625 and MB064 in the BI farm. In general, the CHE samples showed more complex electropherograms with a higher number of stutters and a higher level of background.

The alleles assignment of the BM and CHE samples was carried out several times in separate sessions in order to verify its reproducibility, and consistent results were achieved in the different sessions. However, the loss of one of the longer alleles occasionally occurred.

Not all alleles present in each GO were always detected in the corresponding dairy products, showing a loss of alleles between the genotyping of subjects included in GOs and that of BMs and CHEs. For five markers (AGLA232, BMS0607, BMS2142, MB025, and MB064), the number detected in BM end/or CHE exceeds the number of alleles detected in the corresponding GO, which explains some “penalty score” values.

### 3.4. Evaluation of the Traceability of Dairy Products

The BM and CHE samples showed an STR profile represented by a subset of markers belonging to animals on lactation at the time of sampling (GO). Furthermore, the BM and CHE produced by distinct groups of animals in different months showed different STR profiles when all 20 markers analyzed were considered.

In order to verify the effectiveness of the selected panel and the implemented algorithm in tracing the GO of dairy products, STR profiles of six BMs (three from BI and three from IM) and six CHEs (three from BI and three from IM) were submitted to the algorithm evaluation.

Table 4 shows the penalty score and correlation values obtained by applying, respectively, the verification and the simulation analysis.

The penalty score values were similar for both farms and ranged from 0.06 to 0.20 for BM and CHE of BI and from 0.00 to 0.29 for BM and CHE of IM. Correlation values range from 0.44 to 0.80 for the BI LM and FOR and from 0.44 to 0.75 for the IM samples.

**Figure 1:**
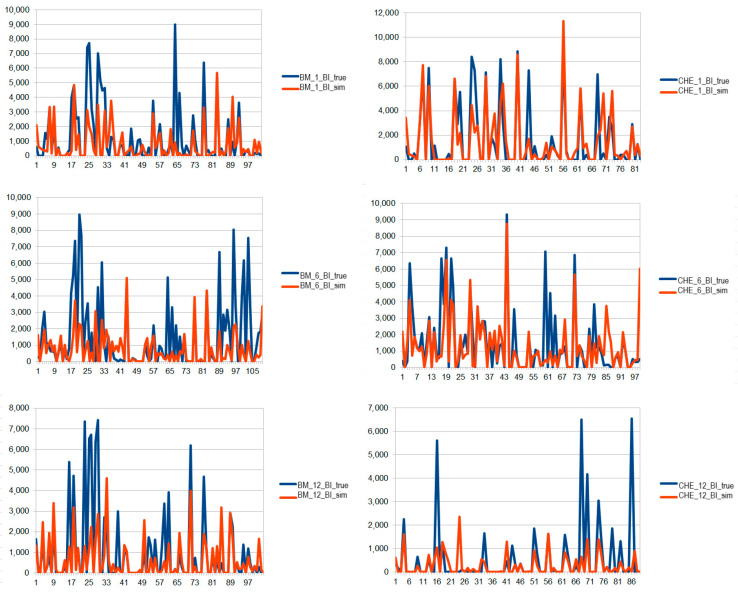
Simulation analysis carried out on the STR profile of the BM and CHE produced by the BI GO. Blue line: combination obtained by the analysis of the sample. Orange: ideal linear combination corresponding to the farm (GO) simulated by the algorithm. On the *x*-axis, the alleles in ordered sequence are displayed; the *y*-axis reports the relative fluorescence unit (RFU) for each allele.

**Figure 2:**
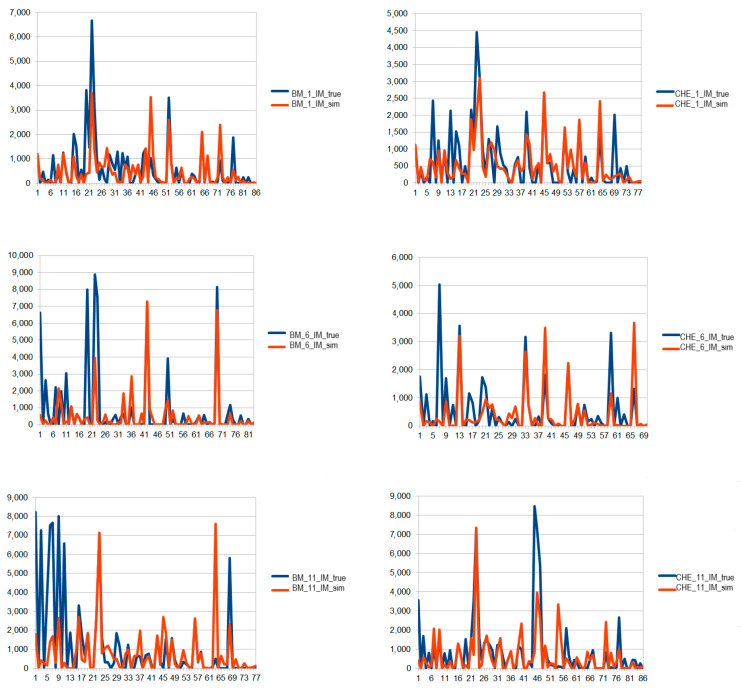
Simulation analysis carried out on the STR profile of the BM and CHE produced by IM GO. Blue line: combination obtained by the analysis of the sample. Orange: ideal linear combination corresponding to the farm (GO) simulated by the algorithm. On the *x*-axis, the alleles in ordered sequence are displayed; the *y*-axis reports the relative fluorescence unit (RFU) for each allele.

Figure 1 and Figure 2 show the graphs of the simulation analysis corresponding, respectively, to the BM and CHE of BI farm and to the BM and CHE of IM farm.

The values of correlation and penalty score obtained by comparing the BM and CHE produced by BI farm with the GO of IM and *vice versa* are reported in Table 5. The penalty score values ranged from 0.54 to 0.86 and the correlation values ranged from 0.10 to 0.54, highlighting a higher genetic distance between the GO signature and the dairy products’ fingerprinting of dairy products derived by different farms.

## 4. Discussion

Improving food traceability is crucial in order to hinder food counterfeiting, to guarantee the quality and the safety of food products, and to safeguard risk categories (i.e., allergic people or intolerant to particular foods or additives).

Chromatographic and molecular methods are the major approaches applied in the detection of food fraud [2]. Molecular approaches show some important advantages such as accuracy, sensitivity, and high reproducibility [9,13]; particularly, “omic” technologies (genomic, transcriptomic, proteomic, and metabolomic) are used for authentication issues in foods of animal origin, providing results of a higher analytical quality [15].

Protein-based, spectroscopy, and chromatographic techniques are also available for the detection of adulteration of dairy products [2,9,14,15,30], and several DNA-based methods are available for species/breed differentiation in dairy products [2,9,16].

The traceability of dairy products with PDO or PGI labels has become an important issue for authentication because they have high commercial and nutritional value and the breeds and geographical origins from which these products are derived have been used as market symbols and have encouraged rural development and breed conservation [16]. DNA-based techniques, metabolomics approaches, and chromatographic methods have also been used for the verification of PDO or PGI products [2,14,15,16,22].

Despite the several molecular methods available, fraudulent techniques continue to diversify and evolve, challenging current techniques for determining food authenticity; therefore, the development of more efficient and reliable molecular tools is necessary [16].

Although primarily for financial gain, in many cases food fraud can have direct and/or indirect consequences on food safety [14]. In this context, cheese produced with milk originating from unauthorized farms (i.e., not checked by the health authority) could cause serious harm to consumers’ health.

Proteome and metabolome have been used for finding an unequivocal and reliable fingerprint to correctly identify species/breed or the geographical origin of dairy products [14,15].

In the present study, we established for the first time a biomolecular method based on the analysis of a panel of microsatellites to identify in bulk milk and in derived cheeses a “DNA fingerprint” of small dairy farms and to assign dairy products to the corresponding producers. Two small Italian farms were involved and a blood and milk sample from each cow, bulk milk, and the deriving cheeses were collected during a period of 12 months. Due to the different organizations in the production method, sampling did not take place simultaneously for the two farms, but at the end of the sample collection period, the expected number was reached.

DNA quality is one of the main factors influencing the dairy products’ authentication by molecular methods, being affected by the manufacturing process, extraction method employed, and chemical composition of the matrix [16]. Among others, the presence of inhibitors, calcium, lipids, proteins, and DNA degradation in processed foods are reported issues in obtaining good yield and quality of DNA when analyzing dairy products [31].

The extraction protocols adopted in our study were effective and suitable, showing that it is possible to obtain DNA of good quality and quantity for the analysis of microsatellites, even from difficult matrices such as high-fat containing milk and ripened cheeses. The difficulty in extracting DNA of good quality from ripened dairy products is known, and indeed, the extraction yield was higher for milk than for cheese. Ripening can reduce the integrity of DNA, thereby affecting DNA amplification detection [16,22]. Rentsch et al. verified that long periods of cheese ripening result in losses of amplifiable DNA compared to fresh cheeses, probably because of DNA degradation [16,32]. Also, the quality of the extracts was higher for the milk samples compared to the cheeses. However, these variations did not produce, in all the samples tested, difficulties in amplifying segments up to 200 bp. Amplification of targets with a length of more than 300 bp displayed some problems, probably due to the greater DNA degradation related to the cheese-making process and ripening, as already reported [10,33]. These results led to the selection of markers shorter than 300 bp and this strategy allowed the amplification of all the samples, except for 5% of BM samples and 6.3% of CHE samples. All the selected markers (except for AGLA29 for the BI GO) showed PIC values of 0.5 or more and can be regarded as highly informative (Table 2) [34].

Tests performed on single milk samples to verify reproducibility and the possibility of replacing blood for individual typing gave positive results, although in three cases only one of the two alleles was amplified at the first run, probably due to the allelic dropout. Therefore, it is advisable to repeat the analysis at least three times (or more in case of doubtful results) when individual milk is used for the typing of subjects to limit the loss of genetic information.

The difference in DNA quantity and purity could explain the slight differences in the number of alleles detected among the GO and the respective BM and CHE [16,22,32]. The descriptive statistics showed good gene variability of the two GOs, with average levels of differentiation between the two groups (Table 2). The dairy products showed a fewer number of alleles than the GO. Considering all of the BM and CHE samples and all markers, a higher number of alleles in CHE than BM was detected in both farms, although always lower than the number of alleles found in the GO (Table 3). In three BM and in six CHE, the number of detected alleles was higher than that detected in the GO (Table 3). These results mainly occurred for two markers (MBS2142 and MB025) and are likely attributable to greater DNA degradation in CHE samples and to the peaks profile of the STRs, where the stutters can be misinterpreted and alleles assigned by mistake. In this context, it needs to be pointed out that STR analysis of BM and CHE samples generates complex electropherograms that must be interpreted by an experienced reader. The knowledge of the alleles found in the GO could have an impact on the interpretation of the electropherograms (reducing the error and leading to distorting the computer analysis), and therefore it was decided not to make this information available to the reader.

As predictable, not all the alleles present in each GO were detected in the corresponding dairy products, showing a loss of alleles between the GO and BM or CHE samples genotyping. This finding may depend both on the intrinsic characteristics of the genotyping by microsatellites (allelic dropout) and on the features of BM and CHE matrices which influence the extraction efficiency and purity of the obtained DNA [16,22,32]. From this point of view, the study has brought new knowledge on STR amplification efficiency in complex matrices (BM and CHE) and on the loss of genetic information compared to the analysis of matrices such as blood and milk.

No qualitative differences in terms of reduction in the method efficiency and of genotyping results were observed in relation to the number of animals included in the GO on the basis of the lactation period of each cow (from a minimum of 4 to a maximum of 14). This allows us to state that the method of analysis developed in this study is not affected by the different numbers of animals involved, at least up to a maximum of 14 cows.

Among the DNA markers studied for traceability purposes in livestock to differentiate breeds, STRs are widely studied but none have been implemented as a tool for breed differentiation or dairy product authentication [16]. Sardina et al. (2015) identified three microsatellites which can be applied for genetic traceability of the Girgentana breed in dairy products in order to detect adulteration due to Maltese and Derivata di Siria goat breeds [35].

Despite their limited use for the traceability and authentication of foods of animal origin, STRs are widely used for agri-food traceability including varietal identification and adulteration detection due to their high reproducibility, polymorphism degree, possible standardization, and straightforward detection systems [13].

Our results showed that the STRs profile detected in BM and CHE is composed of a subset of markers among those existing in lactating animals at the time of the collection. Furthermore, BM and CHE produced in different months showed different STRs profiles. In some cases, a difference in a few alleles among the GO, BM and CHE was observed; indeed, alleles not included in the corresponding GO have been occasionally detected in CHE and BM. Nevertheless, those alleles could be of cows hosted in the farm of origin (but not producing milk in the sampling period); therefore, we supposed a low level of contamination by equipment or instruments. Overall, the farm of IM and BI showed variable allelic lengths, with some shared alleles and others characteristic of each farm, which explain the Fst value (Table 2). These data are easily explained since the two stables are made up of animals of different breeds and belonging to distinct geographical areas. Despite these genetic differences, a deterministic approach is not efficient in excluding a GO with respect to a product (BM and CHE) coming from the other farm.

Considering the two farms separately and applying the algorithm implemented by Rossi et al. (2018) [29], satisfactory results were obtained. The penalty score values between 0.0 and 0.35 (on a scale of 0–1) lead to not exclude the GO for each BM and CHE considered, and therefore, they recommend continuing the simulation analysis. For some samples, the penalty values are different from 0 due to the presence of the “contaminating” alleles, likely due to a false allele attribution due to the presence of stutter peaks, which led to identify stutter peaks as true alleles.

The correlation values from 0.44 to 0.75 highlight the existence of a correlation between the GO signature and the fingerprinting of the corresponding BM and CHE products, although the data are also influenced by the presence of contaminated alleles.

The test performed by crossing the data obtained by BM and CHE of the IM GO with those of the BI farm, and *vice versa*, highlights higher penalty score values (0.54–0.86), indicating little compatibility of the products with the GO. In this simulation, the correlation values are instead low (0.10–0.42), pointing out the absence of linear correlation between GO and dairy products and supporting an exclusion of the GOs as producers of the dairy products. In Rossi et al. (2018) [29], we reported the correlation analysis between the GO with a 50% and 100% “fraudulent” BM and CHE (i.e., with 50% and 100% cows not belonging to the declared farm), showing that the difference among the three groups (100% authentic, 50% fraudulent, and 100% fraudulent) was statistically significant (*p* < 0.05).

Therefore, our study developed a protocol of analysis with a selected panel of highly informative STRs and demonstrated the effectiveness of a tool combining the molecular analysis of those STRs markers and the informatic evaluation based on a heuristic algorithm for the genetic traceability of dairy products.

The proposed protocol shows many advantages of DNA-based approaches for dairy products traceability: sensitivity, repeatability, and reliability [9,15,16,22]. The protocol can be applied to processed dairy products like ripened cheese and can be multiplexed, lowering the costs and time of analysis. Moreover, compared to other molecular methods like Next Generation Sequencing (NGS) and Single Nucleotide Polymorphism (SNP), it does not require high-quality DNA and bioinformatic skills [10,13,22].

One of the problems related to DNA-based approaches for food authentication is the lack of procedure standardization and universality [15,16]. The protocol we presented poses the same problem, considering the susceptibility of the results to sample preparation methods and to different interpretations of the electropherograms. In our opinion, these issues could be overcome using NGS instead of capillary electrophoresis.

Although the implemented method can be improved by entering as input data the relative quantities of each detected allele, it provides numerical values, namely penalty scores and correlation values, which are comparable and evaluable from a statistical point of view and useful for the traceability of the dairy products. In fact, these values could be useful mainly in assessing the probability that a traditional dairy product does not come from a production farm (exclusion approach) and therefore in providing a measure of the level of potential counterfeiting.

Given the need to genotype all the cows in the GO, the method we present is not suitable for use in routine test, but it could be a helpful tool to verify suspected fraud, particularly for traditional dairy products produced by small farmers. Based on the results obtained, an applied study should be carried out on a larger scale, i.e., on a greater number of farms and/or farms with a greater number of lactating cows than that of the farms recruited in this project; the ultimate aim should be the availability of a tool which is useful to producers and control authorities both for product certification and for fighting against fraud.

## 5. Conclusions

Food fraud has become a global problem, and prevention is increasingly the focus of producers and government authorities responsible for ensuring food quality and safety. Among the most adulterated foods are milk and dairy products, mainly due to the nutritional value of milk, the global demand, the reduced shelf life, and the lack of new methods for authenticating these products.

Although there are several molecular methods for the detection of adulterant species and the authentication of the breed from which the foods are produced, there is still an unmet demand for techniques to trace dairy products back to the farm of origin.

Here, we developed for the first time a molecular method, based on the analysis of a panel of microsatellites, to assign milk and cheese to the corresponding producers in the context of food traceability and counter-fraud measures.

Our study allowed the development of a DNA extraction and amplification protocol that can be applied to high-fat and processed food. We identified a panel of 20 highly informative markers and demonstrated that dairy products expressed an STRs profile composed of a subgroup of STRs belonging to the animals from which the dairy product originated.

The panel of STRs and the implemented algorithm proved their usefulness in the traceability of dairy products obtained from small producers by the assessment of the correlation between the genetic fingerprinting of the product (BM or CHE) and the genetic characteristics of the GO.

Considering the constant demand for the development and improvement of analytical methods, our study represents an innovative approach, although improvable, which becomes part of the range of techniques that can be used to prevent food fraud.

## Figures and Tables

**Table 1:** STRs were selected based on size, polymorphism, and lack of linkage disequilibrium reported in Brown Breed (BB). Location on Bovine Genome chromosome (BTA) and primer sequences are reported. STRs labelled with a star were selected and included in the final panel of analysis.

MARKERS	BTA	SIZE	N° Alleles in Brown Breed	Forward Primer Sequence	Reverse Primer Sequence
DIK5019	1	189–205	6	TTGTACGTCTCTGGGGCTGT	CTGACCCCCATGCTTTAAGA
MB108	1	248–274	5	GGACACGACTGAGCAAGTAA	AGGCAGATACATTACCACTA
DIK2396	1	199–222	4	CTGGGAAGAACCTGACGTTT	GTTGGGAAGATACCCTGACG
BMS4008	1	150–176	5	CGGCCCTAAGTGATATGTTG	GAAGAGTGTGAGGGAAAGACTG
DIK104	1	120–150	6	TTAACATGGTGTGAACCCAC	CATAAAGTCTTCCAGGTCTC
CSSM019	1	137–155	5	TTGTCAGCAACTTCTTGTATCTTT	TGTTTTAAGCCACCCAATTATTTG
BMS918	1	133–143	4	AGTCTTCTCTGACAGCAGTTGG	CCAGGTACCAGAGAGAGGAGA
BMS4044 *	1	132–162	5	TGGATTCTTAACCACTGGGC	TTTCCTTACATTTTGACTGTCCC
TGLA431	2	134–163	5	GGTCATATCCTTCAAAATTTACTTA	CCCATTTATTCCTAATTTCAACTTC
TEXAN04	2	110–136	5	AGAAGTCCTTGCTTTCACAGAC	TTTTGGTGCTTGATGCTATTAG
BM2113	2	123–143	5	GCTGCCTTCTACCAAATACCC	CTTCCTGAGAGAAGCAACACC
INRA006_MB101	3	106–120	4	AGGAATATCTGTATCAACCTCAGTC	CTGAGCTGGGGTGGGAGCTATAAATA
BMS0963	3	134–154	6	GGAGGATGAAGGAGTCTTTGG	AATTTACCACAGTCCACCGC
HUJII77	3	187–213	5	TCCATCAAGTATTTGAGTGCAA	ATAGCCCTACCCACTGTTTCTG
BMC4214 *	3	172–202	7	CTAGATTGTTTTCTATGAACAGGGG	GCATTTCCAGACCTTTCCTG
RM188	4	123–147	5	GGGTTCACAAAGAGCTGGAC	GCACTATTGGGCTGGTGATT
RM088_CA088	4	124–150	5	GATCCTCTTCTGGGAAAAGAGAC	CCTGTTGAAGTGAACCTTCAGAA
INRA133 *	6	206–232	5	ATCCTCAAAGCAACCTGGC	GAATCTTCTCCCCCTGCATC
BM0143 *	6	90–118	5	ACCTGGGAAGCCTCCATATC	CTGCAGGCAGATTCTTTATCG
BM9065	7	162–184	5	ACTCTCCCTCCACACAGGG	GTCAACCTCAGCAAAACTGATG
ILSTS006_MB057	7	281–299	5	TGTCTGTATTTCTGCTGTGG	ACACGGAAGCGATCTAAACG
BMS1247	7	103–129	5	TCAGCTCTCAGCAGCCTGTA	GGGGTTAATGGTGATCTGCA
DIK2633	8	225–251	4	TGAACTGAAAGCCATGAGAGTG	TCTGGAGGCTGGGAAATCTA
BL1080	8	119–143	6	TTCTGAATGCACCCTTGTTTAG	CTGGGCAACTAACTAATCCTGG
DIK4508	8	188–203	4	TTAAAAATCCCTGGGCAACA	GCAGAGGTATTGGCTTTAGCA
DIK671	8	123–149	8	TGGTGGAAGACTGATGGTCA	GCCATTTGGACCTATTGAGG
Z27077_idvga11 *	8	103–123	6	CCTCTGGGTCTATCCATGTTG	TGGATGAATGAAGAAGATGCC
SRC276 *	8	181–197	5	GCCTATGACATTGCTTATGA	CCTATAGTGGAATGTAGCAG
BMS0607 *	11	133–169	6	ACAGCCTCTGGGACCAGTC	GATAACCGCATACAAACTGGC
BM720 *	13	210–240	7	ACATCTCATTCTTGTGTCATGG	GAAATTGACTTTAGGGTTCCCC
URB58_URB021B	13	176–188	6	GTAAGGCTCTTTGAGGGTTAGG	GCTTAGAAGTTTCTGTGCTGTC
AGLA232 *	13	155–183	7	CCTTTGCAAATACCTCCTGACCAG	AATGGTTCTACATTTGCTAGGTGTC
CSSM066	14	177–197	6	ACACAAATCCTTTCTGCCAGCTGA	AATTTAATGCACTGAGGAGCTTGG
BMS947	14	112–138	8	GTGGGAGTTACTTCCTTCTGTTT	TGGGAACACTATGATTTTCTCC
BL1036	14	178–202	5	TAGCTTATGCCATTGTTTTTGC	ATCTGATGTGGGTTTCTGACTG
BMC1207 *	14	128–152	6	ACCAACAAGTCTGAATCTTCATT	GGGTGGAATAGTCAGTCCCA
RM004_CA004	15	113–129	6	CAGCAAAATATCAGCAAACCT	CCACCTGGGAAGGCCTTTA
MB064_HBB *	15	150–168	7	GGGACTCATAGACCATTCATAGC	CAACTGGCAAAATTTCATTCTT
BM1706 *	16	233–259	5	ACAGGACGGTTTCTCCTTATG	CTTGCAGTTTCCCATACAAGG
HUJ625 *	16	196–224	7	AGCAGCATGAAGAGAGTCCC	GAGGTCACATACCCATCAAGC
CSSM028	16	142–172	6	TTACTGAAGATCCCTTCTAATGAG	GATAACAGTGTCTCATCAAATACA
BMS499	17	102–130	6	CAGGCTTAAGTATCAAACTTTCTTC	TTTAAGGTAGATGGGTAGTTGTACG
ETH185_MB008	17	222–243	5	TGCATGGACAGAGCAGCCTGGC	GCACCCCAACGAAAGCTCCCAG
BM2078	18	92–114	4	CCCAAAAGAAGCCAGGAAG	TCAGAGTTTGGGGTCCTCAG
BMS2142 *	19	91–113	7	AAGCAGGTTGATGATCTTACCC	GTCGGCACTGAAAATGATTATG
AGLA29 *	20	144–166	6	AGGAAGCCGAGTGAGATATGTAAGC	TTACAGCCTGTGTGAATGTCCTCTA
TGLA126	20	116–122	3	CTAATTTAGAATGAGAGAGGCTTCT	TTGGTCTCTATTCTCTGAATATTCC
ILSTS072	20	157–177	6	ATGAATGTGAAAGCCAAGGG	CTTCCGTAAATAATTGTGGG
TGLA122	21	137–181	4	CCCTCCTCCAGGTAAATCAGC	AATCACATGGCAAATAAGTACATAC
HEL05_MB071 *	21	147–165	6	GCAGGATCACTTGTTAGGGA	AGACGTTAGTGTACATTAAC
RM185	23	96–112	7	TGGCCTGCTTATGCTTGCATC	GAGTTTCCTTTGCATGCCAGTC
AGLA291	23	261–277	6	TTCTCTCAAATGATGAATATGCTTC	GGACTATTCTATGCATGCCTCTC
MB026	23	202–229	5	GGACACGTTCTGCAGATACAACTAC	GAACTCTCCTTAAGCATACTTGCTC
MB019	23	188–224	6	GGAGGGTTACAGTCCATGAGTTTG	TCGCGATCCAACTCCTCCTGAAG
MB025 *	23	121–137	5	ATGGTGCAGCAGCAAGGTGAGCA	GGGACTCAGTCTCTTATCTCTTTG
RM185	23	96–112	6	TGGCCTGCTTATGCTTGCATC	GAGTTTCCTTTGCATGCCAGTC
BMS1353	25	90–130	6	TTTCAGGACTAATAGGGCATGG	ATTCAGACCTGCCTGGTGAC
BMS651	26	109–145	6	AATATGTGAAAACAAGTCAAAGCA	CCTGGCAAGCAACAGTTAAT
INRA134	27	97–147	7	CCAGGTGGGAATAATGTCTCC	TTGGGAGCCTGTGGTTTATC
BM3507 *	27	159–189	6	GCCCAAAGAAAGAAGTATGTGC	TAGTGCGGAGTCAGTCATGTG
BMC6020 *	28	157–203	7	ATTGCATGTAGCTCTTGGGG	AAGTGGGTGGCTTCAACACT
BM4602 *	29	112–144	7	GTGCATTCACACATCTCCATG	GCAGCTTTAGCATCTGGGTC

**Table 2:** Descriptive statistics obtained by the genotyping results of the cows included in the two GOs. The number of alleles, gene diversity and observed heterozygosities, Fis and Fst values per locus and population are reported. In the last two columns, the informativeness of each genetic marker is indicated by the polymorphism information content (PIC) values.

	Number of Alleles			Gene Diversity per Locus and Population		Observed Heterozygosities		Fis		Fst	PIC Values	
Marker	BI	IM	Total	BI	IM	BI	IM	BI	IM		BI	IM
BM3507	6	10	11	0.822	0.909	0.917	0.786	−0.115	0.136	0.009	0.760	0.859
BM4602	6	7	8	0.799	0.799	0.750	0.786	0.062	0.017	0.024	0.731	0.738
BMC421	5	7	7	0.792	0.577	0.833	0.571	−0.053	0.010	0.036	0.721	0.530
BMS214	5	5	6	0.761	0.703	0.750	0.643	0.015	0.086	−0.001	0.684	0.625
INRA13	6	5	7	0.674	0.382	0.833	0.357	−0.236	0.065	0.040	0.602	0.348
BM0143	5	7	8	0.784	0.797	0.750	1.000	0.043	−0.255	0.012	0.706	0.745
BM1706	6	7	8	0.792	0.824	0.750	0.714	0.053	0.133	0.009	0.722	0.761
BMC602	6	7	8	0.602	0.860	0.500	0.929	0.170	−0.080	0.076	0.544	0.810
BMS404	7	7	10	0.867	0.777	0.833	0.643	0.039	0.173	0.036	0.807	0.715
MB025	5	7	8	0.731	0.780	0.833	0.786	−0.140	−0.007	0.076	0.656	0.719
AGLA23	7	10	10	0.830	0.890	0.750	0.714	0.096	0.198	0.031	0.763	0.836
HUJ625	8	7	8	0.879	0.808	0.833	0.714	0.052	0.116	0.003	0.821	0.750
MB064	6	6	7	0.833	0.712	1.000	0.857	−0.200	−0.205	0.042	0.778	0.660
Z27077	8	6	10	0.795	0.841	0.750	0.714	0.057	0.150	0.053	0.734	0.777
MB071	7	3	7	0.799	0.610	0.917	0.500	−0.147	0.180	0.012	0.741	0.498
BM720	6	10	10	0.807	0.876	0.917	1.000	−0.136	−0.141	0.015	0.745	0.833
BMS060	7	10	13	0.754	0.901	0.833	0.846	−0.106	0.060	0.068	0.683	0.850
SRC276	7	4	7	0.727	0.772	0.917	0.714	−0.260	0.075	0.010	0.666	0.694
BMC120	4	6	7	0.667	0.709	0.583	0.643	0.125	0.093	−0.003	0.573	0.644
AGLA29	3	5	6	0.485	0.717	0.250	0.857	0.484	−0.195	0.038	0.410	0.646
TOTAL	120	136	166	0.76 ± 0.021	0.76 ± 0.028	0.77 ± 0.027	0.7387 ± 0.026	−0.020 *p* = 0.748	0.031 *p* = 0.142	0.058 *p* = 0.00020		

**Table 3:** Results of the analysis of BM and CHE. For each marker and each farm, the number of alleles detected in the group of origin (GO) and two different matrices (bulk milk and cheese) are reported.

	BI Farm			IM Farm		
	GO	BM BI	CHE BI	GO	BM IM	CHE IM
AGLA232	7	7	8	10	7	8
AGLA29	3	3	3	5	5	4
BM0143	5	4	5	7	7	6
BM1706	6	4	4	7	5	6
BM3507	6	6	6	10	8	8
BM4602	6	6	6	7	6	6
BM720	6	6	5	10	10	10
BMC1207	4	3	4	6	4	5
BMC4214	5	5	5	7	3	4
BMC6020	6	5	6	7	7	7
BMS0607	7	5	6	10	9	12
BMS2142	5	6	6	5	6	6
BMS4044	7	7	5	7	6	5
HUJ625	8	8	8	7	7	7
INRA133	6	4	6	5	5	5
MB025	5	6	7	7	6	5
MB064	6	6	8	6	6	6
MB071	7	7	7	3	3	3
SRC276	7	5	7	4	4	4
Z27077	8	6	7	6	5	5
Total	120	109	119	136	119	122

**Table 4:** Values obtained from verification and simulation analyses carried out with the developed algorithm on each GO and three of the respective LM and FOR.

BI Farm			IM Farm		
	Correlation	Penalty Score		Correlation	Penalty Score
**BM_1_BI**	0.62	0.17	**BM_1_IM**	0.68	0.21
**CHE_1_BI**	0.80	0.20	**CHE_1_IM**	0.75	0.00
					
**BM_6_BI**	0.58	0.11	**BM_6_IM**	0.65	0.00
**CHE_6_BI**	0.70	0.35	**CHE_6_IM**	0.61	0.08
					
**BM_12_BI**	0.64	0.06	**BM_11_IM**	0.44	0.00
**CHE_12_BI**	0.44	0.12	**CHE_11_IM**	0.66	0.29

**Table 5:** Values obtained from verification and simulation analyses carried out with the developed algorithm performed on each GO and the BM and CHE of the other GO.

	CORR	PENALTY
BI BM vs. IM GO	0.348	0.86
BI CHE vs. IM GO	0.424	0.75
IM BM vs. BI GO	0.106	0.57
IM CHE vs. BI GO	0.176	0.54

## Data Availability

The data presented in this study are available upon request from the authors.
